# On the origins and evolution of qualia: An experience-space perspective

**DOI:** 10.3389/fnsys.2022.945722

**Published:** 2022-08-10

**Authors:** Thurston Lacalli

**Affiliations:** Department of Biology, University of Victoria, Victoria, BC, Canada

**Keywords:** qualia, phenomenal experience, evolution of consciousness, E-space, dimensional sorting

## Abstract

This paper elaborates on a proposal for mapping a configuration space for selector circuits (SCs), defined as the subset of neural correlates of consciousness (NCCs) responsible for evoking particular qualia, to its experiential counterpart, experience-space (E-space), as part of an investigation into the nature of conscious experience as it first emerged in evolution. The dimensionality of E-space, meaning the degrees of freedom required to specify the properties of related sets of qualia, is at least two, but the utility of E-space as a hypothetical construct is much enhanced by assuming it is a large dimensional space, with at least several times as many dimensions as there are categories of qualia to occupy them. Phenomenal consciousness can then be represented as having originated as one or more multidimensional ur-experiences that combined multiple forms of experience together. Taking this as a starting point, questions concerning evolutionary sequence can be addressed, including how the quale best suited to a given sensory modality would have been extracted by evolution from a larger set of possibilities, a process referred to here as dimensional sorting, and how phenomenal consciousness would have been experienced in its earliest manifestations. There is a further question as to whether the E-space formulation is meaningful in analytical terms or simply a descriptive device in graphical form, but in either case it provides a more systematic way of thinking about early stages in the evolution of consciousness than relying on narrative and conjecture alone.

## Introduction

Much of the explanatory success of the scientific enterprise flows from the power of the reductionist enterprise, where a phenomenon is understood by investigating the structure and dynamics of subcomponents of which it is constructed. This methodology has long since proven its utility where those subcomponents have a material existence and behave in ways that can be observed and measured, whether stars and planets or atoms and quarks. It is problematic when we come to investigate consciousness, whose subcomponents, the contents of consciousness, are neither material in nature nor assignable to a specific spatial location. The most intractable issues, the hard problems of consciousness, relate to the nature of phenomenal experience and its physical source (Levine, 1983, 2009; Chalmers, 1995). However, from a developmental perspective, there is a more prosaic problem of explaining how the neural circuits responsible for generating and/or evoking such experiences are correctly assembled in the embryo. I examined this issue in a preliminary way in an earlier paper (Lacalli, 2020) that explored how Alan Turing’s ideas about the emergence of pattern during development might be applied to explain the emergence of consciousness during evolution. Only questions concerning weak emergence (*sensu* Bedau, 1997) can be addressed by this means, which restricts the analysis to the proximate physical correlates and determinants of subjective experience (here, by convention, simply “experience”), meaning the assembly of the relevant neural circuitry. The problem of emergence at the material level is then solved, at least in principle: that given the random variations in circuitry and neural activity that inevitably arise during brain development, the reordering required for consciousness to emerge from the preconscious condition is a matter of having mechanisms in place to selectively amplify those few variants that incrementally move the system toward consciousness. The process as a whole can be characterized as the extraction of order from fluctuations across time scales, because amplification occurs both in real time during development, and across evolutionary time through changes in gene frequencies.

The analysis was extended in a second paper (Lacalli, 2021) on a specific subset of neural correlates of consciousness, namely the selector circuits (SCs) responsible for evoking a particular experience rather than some other, to better understand how SCs behave in response to natural selection. SCs are equivalent in this usage to difference makers of consciousness (DMCs, Klein et al., 2020; see also Hohwy and Bayne, 2015), and are less neutral in a causal sense than the broader category of NCCs (Neisser, 2012), that is, they are more than just correlates. And, it should be pointed out, that so long as consciousness is assumed to be a consequence of neural activity, the DMC/SC formulation is valid regardless of what theory of consciousness one adopts. That is, even for higher order theories that take a representational view of consciousness, that it resides in the algorithmic processing of neural input in and of itself (Van Gulick, 2018; Lycan, 2019; Seth and Bayne, 2022), there will necessarily be components of brain circuitry, whether localized or distributed diffusely across cortical networks, that govern the precise form of experience evoked by a particular sensory input. A configuration space representation is then a useful way of exploring how the constraints on SCs for the simplest of conscious contents change over evolutionary time. How a configurational, neurocircuitry-based SC-space might map to an experiential space (E-space) is a separate issue, and there are no clear guidelines as to how best to construct such a space, what its dimensions represent, or how many there might be. Here, to investigate such questions, the utility of E-space as a conceptual tool is explored further, with attention to the problem of representing diverse qualia in spaces of more than two dimensions.

This is not intended as a rigorous topological exercise, nor it seems, can it be, for reasons discussed below. Instead it is at this stage simply an investigation of a particular graphical construct as a tool for dealing conceptually with how phenomenal consciousness would unfold over evolutionary time in response to changes at the level of the SCs. Based on the ideas of von Békésy (1959), one can draw provisional conclusions regarding the nature of at least one E-space dimension: that among the properties to which mechanosensory qualia map (here combining tactile and acoustic experience), one of these properties will be time-related. More importantly, the analysis provides insights into the nature of ancestral experience prior to the emergence of a more differentiated form of consciousness, making the case that if evolution is to assign qualia to sensory modalities in an optimal way, the best starting point is to have ur-qualia that are diffuse and extend through many dimensions. A sorting process will then follow whereby different categories of qualia are progressively restricted to non-overlapping domains (i.e., exclusive sets of dimensions) in E-space. This provides insight into otherwise problematic issues, including how consciousness might have been experienced at different stages in its early evolution.

## Exploring experience-space: Dimensionality and time

**FIGURE 1:**
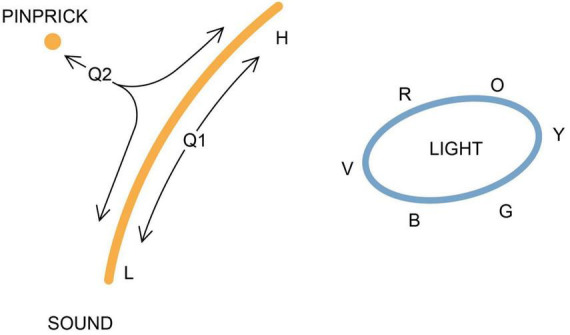
An experience space (E-space) representing three kinds of qualia in two dimensions, modified from Lacalli (2021). A pinprick, among the simplest of tactile experiences, and disregarding its localization, would be a point. Sound, for animals that can consciously distinguish pitch, would be a set of related qualia ranging from low (L) to high (H) pitch. Color, as we experience it, would be a closed curve, as the sequence from red to orange, yellow, green, blue, and violet (R, O, Y, G, B, V) is recursive, leaving the center of the curve for their blended combination, white light. The trajectories (arrows) show possible evolutionary sequences: that the pitch range of acoustic experience could originally have been limited to an acoustic ur-quale at Q1, and then have expanded over time; or that both tactile and acoustic sensations could have a common origin in an intermediate ur-quale (at Q2) that combined features of both, making the descendant qualia homologous as experiences (see Lacalli, 2022 for further comment on homology at the experiential level). The SCs responsible for evoking intermediate sensations along the trajectories will have been extinguished by selection, but will have existed in the past whereas, for qualia unrelated through homology, there may be no such intervening points, and hence no access to intermediate experiences. This may be the case for light and mechanosensations, which share no obvious qualitative features, in which case there would be no justification for mapping them to the same surface.

E-space (Figure 1) is designed to be an experiential counterpart to my configuration space representation of SCs. It was conceived as a way to map the qualitative properties of phenomenal experience so as to reflect the logic of how SCs influence that experience, so that axes in E-space would correspond to some combination of the neural features and/or events that characterize SC space. In this sense, there is no implied dualism, that E-space in some way represents a virtual realm separate from the material world. It is, instead, simply a mapping. And, as with SC-space, it applies only to qualia, conceived of as fundamental units of experience, and hence is unsuited for representing more complex contents that depend on sequential processing at a neurocircuitry level. So, for example, light perception can in principle be investigated using E-space, but not the visual display as a total experience.

E-space is constructed also to be ontologically fixed, in that it maps all qualia that could potentially exist in consequence of neural activity, whether experienced by any particular brain or not. As such, it represents a fixed domain of possibilities that evolution explores through neural innovation, encountering qualia of adaptive utility in much the same way that an exploration of the various mineral elements available in sea water would identify calcium as the one most suitable for constructing shells and skeletons. E-space therefore differs from topological constructs used to map empirical data on conscious experience based on subjective reporting, including similarity space (Raffman, 2015) and quality space (Rosenthal, 2015), which are, in any case, not designed to address the problem of evolutionary change. And, though subjective reporting is used here as a guide to constructing the figures, e.g., in the choice of acoustic pitch and visual hue as variables for mapping, this choice is provisional, and may require revision once data are available on real SCs, as opposed to hypothetical ones, and the way they map to experience. The main conclusions of my analysis are, in any case, of a general nature, and valid irrespective of the specific details of how E-space axes are defined.

Two mechanosensory modalities are included in Figure 1, a pinprick, to represent sharp pain, and sound, along with the perceived spectrum of light. These are chosen to provide two separate demonstrations of why E-space must have at least two dimensions. For mechanosensations, this is because deriving both tactile and acoustic qualia from a common ancestral ur-quale requires divergence along two trajectories, which then define separate axes in E-space, one of which can be provisionally assigned to represent pitch. Assuming the range of perceived pitch has expanded over evolutionary time, a trajectory would then be traced out approximating that shown in the figure, beginning at the point (Q1) representing the ancestral acoustic ur-quale. A step further is to suppose a degree of homology between acoustic and non-acoustic mechanosensations, and derive both from an ur-quale (Q2) intermediate between them that combines features of both. This yields a second, independent axis, and the points traced out by this divergence then define a surface of two dimensions at a minimum that, assuming evolutionary change is incremental, is locally continuous. Intermediate points along such trajectories can then be considered real, i.e., they exist, because they have existed in the past in real brains.

A digression is required here on terminology, as to what points in E-space represent. Since E-space is designed to map qualia conceived of as fundamental units of experience, there is a potential problem in supposing they can be assigned subsidiary properties like pitch. The solution to this problem is to treat each point in E-space as representing a single quale, and the subsidiary “properties” as labels that define the relation between a given quale and its close neighbors. The curves and lines in the figure representing modes of sensory experience (sound, light, etc.) are then sets of related qualia, and the domains they occupy (the points, lines and curves in the figure, which can be diffuse or compact) are point clouds that map these sets of qualia. However, to be consistent with previous usage (in Lacalli, 2021), I will use the singular “ur-quale” to refer to ancestral ur-experiences conceived of as point clouds that may combine in one experience the properties of what we would identify as belonging to distinguishable qualia.

**FIGURE 2:**
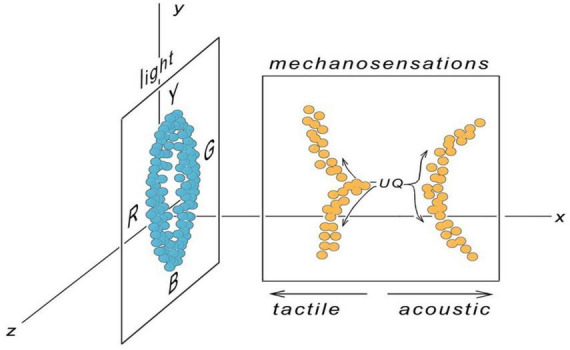
The E-space from Figure 1 reconfigured for 3 dimensions, with mechanosensations and light qualia each occupying their own 2D surface oriented in one of many possible ways. Experiences are shown as point clouds, less compact than in the previous figure, as a reminder that each point in E-space represents the action of a selector circuit (SC) responding to a particular sensory stimulus. The overall density of points is a reflection of how many SCs are dedicated to each sensory modality, and would undoubtedly, for a fully evolved consciousness, be much greater than shown. Here, strictly as a thought experiment, acoustic and tactile sensations are supposed to originate from a common ur-quale (UQ), and share a common axis representing pitch (the *y* axis) in accord with the ideas of von Békésy. In addition, the experience of pain (the pinprick in Figure 1) has been expanded so as to encompass both sharp and dull pain along the same axis as acoustic pitch. Orienting the light plane along this same axis would imply that the yellow/blue axis is pitch-like in some way, while the red/green axis is not. But trajectories along the tactile/acoustic axis intersect the plane for light experience, implying a link between these. As discussed more fully in the text, such implied relationships are difficult to justify, yet are an inescapable feature of mapping more than one category of experience to a space with too few dimensions. Allowing E-space to have more dimensions, perhaps many more dimensions, avoids this problem.

Light perception is shown in Figure 1 as two-dimensional because mapping the recursive feature of light experience, where hues blend into each other to form a color wheel, also requires a minimum of two dimensions. This is adjusted in Figure 2, so the two defining axes are yellow/blue and red/green in accord with current theory (Matthen, 2020), but the question of more immediate concern is whether it is appropriate to represent light qualia on the same surface as mechanosensations. The alternative is for E-space to be multidimensional where n, the number of dimensions, is large (i.e., it is a large dimensional space, potentially with at least several times as many dimensions as there are categories of qualia to occupy them), in which case we have a formal construction that is more difficult to illustrate but far richer in what it can be used to represent. And, since the dimensions are simply hypothetical axes along which the separable properties of experience are mapped, in other words the degrees of freedom for the system, there is no reason *a priori* to limit to their number. This also means dimensions in E-space will differ from those of normal 3D space in that continuity across them is not guaranteed so that, with reference to Figure 1, there may be no route by which a light experience can transition incrementally into a mechanosensation. Assigning qualia to separate sets of dimensions avoids this problem, but there could still be discontinuities between dimensions, which is an impediment to investigating E-space as a whole using mathematical tools requiring continuity.

Before considering arbitrarily large dimensional spaces, a further digression is useful on the problems that arise from having too few dimensions. This is illustrated in Figure 2, which expands the first figure from two to three dimensions. Mechanosensations and light qualia are now represented as restricted to separate planes, with the pinprick-related (tactile) trajectory extended along the *y* axis, so that sharp and dull tactile experiences diverge from a common origin along an axis parallel to that for acoustic pitch. This accords with the classical proposal by von Békésy (1959; 1960; see also Tonndorf, 1986; Manley et al., 2012) that longer wavelength components in the stimulus, whether for sound or mechanosensations more generally, correspond to sensations that are lower pitched and spatially less focused. The *y* axis in the figure would then be a measure of something related to wavelength and frequency, i.e., time, which would not mean time itself, as in the duration of the experience, but some other time-dependent feature encoded in neural activity. Von Békésy’s proposal is useful for illustrating the point that axes in E-space are most easily understood when we have at least a provisional idea of the neural basis for positional shifts along those axes. Yet in most cases this will not be even remotely the case, as to the neural basis of the difference between the sensation of red and green, or yellow and blue, for example, and whether differences along the axes defined by those hues depend on related neurocircuitry features or not.

Consider now what happens if we try to use the pitch axis (the *y* axis in Figure 2) for another set of qualia, namely light perception. The two planes, for mechanosensations and light, could in principle be oriented in various ways in a three-dimensional space, but the point is made by examining two cases, where the planes are either perpendicular or parallel to one another. Take first the perpendicular case, shown in the figure, with mechanosensations and light mapped to planes aligned along the *xy* and *yz* axes, respectively. This implies that both share similar properties across the *y* axis, but otherwise not. As drawn, the shared axis relates acoustic pitch to the yellow/blue axis for light, which would imply that there is something intrinsically “higher pitched” about yellow as compared with blue, but also that this same property could not be used to distinguish red from green. This privileges one set of hues over another, as being more sound-like, which begs the question of how likely it is that distinctions applicable to one sensory modality (here, high vs. low pitch) will apply to others. There is first the problem of separating the quality of an experience from its intensity, for example, in the case of affect (see Cabanac, 2002), whether a strongly felt emotion is one that is more narrowly focused in a pitch-like sense, or simply more intense. At the level of SCs, differences in intensity might simply be a matter of circuit redundancy, with intensity increasing in proportion to the number of SCs available for activation. But consider hedonicity, another of Cabanac’s properties: does that define an axis shared between non-homologous contents, so that a pleasant odor and a sense of contentment might be supposed to depend on a common mechanism at the level of SCs, or not? Though the idea that it does may have some appeal, comparisons of this kind between non-homologous contents have an intuitive component conditioned by the language we use to describe experience that may be quite misleading (Walker et al., 2012; Van Leeuwen et al., 2015), which for my analysis makes the issue as a whole sufficiently problematic that it is better deferred. So, returning to the figure, note the further problem that trajectories along the tactile/acoustic axis for mechanosensations (the *x* axis) intersect the plane representing light experience, implying that whatever separates tactile experience from sound, the more you have of it in one direction or the other (depending on whether the mechanosensory plane is rotated around the *y* axis, or not), the closer you get to a light experience. Absolute distances in E-space are not specified, and very large distances could conceivably account for apparently dissimilar experiences being related in this way through a shared dimension, but it is still a stretch to suppose that a transition through incremental steps is possible between experiences as different as sound and light.

The case of parallel planes can be visualized by rotating the plane for mechanosensations in Figure 2 by 90 degrees along the *y* axis, so it parallels the light (*yz*) plane, but at a different values *x*. The time-related *y* axis is still shared, but now, along the *z* axis, differences between tactile and acoustic experience and red versus green hues would depend on the same property, meaning pain would differ from sound in the same way red differs from green. This is rather puzzling, because differences between yellow and blue are still shown as being frequency dependent, i.e., pitch-like, whereas there is no obvious difference between the experience of yellow vs. blue compared with red vs. green to suggest they differ fundamentally in this way. In sum, the mental gymnastics required to fit diverse sets of qualia into a small dimensional space raises more questions than it answers. The alternative, a more fruitful approach in my view, is to assume E-space extends across many more than three dimensions, and further, that few if any of these dimensions are shared between different categories of qualia as we experience them, as components of a fully evolved consciousness. How this situation would have evolved is a separate question, explored in the next section using the perception of light as an example, to argue for the operation of an exclusionary principle that facilitates both the divergence of qualia and their optimization for particular functions.

## Large dimensional spaces: Light perception and the case for dimensional sorting

Light experience recommends itself to dimensional analysis because its recursive property cannot be represented in less than two dimensions. There is a long history of speculation on color perception, dating to Newton, but current thinking (Raffman, 2015; Matthen, 2020) explains the range of unique hues we experience as arising from the interactions between two principal color axes, yellow/blue and red/green, with a third for white vs. black. The subtleties of how hues are distinguished today is not, however, especially relevant to the evolutionary question of how this mode of color perception originated, because what then matters in biological terms is the ability to consciously distinguish light from the absence of light and from other forms of experience. And, while it is a valid evolutionary question to enquire whether conscious perception of light preceded the evolution of the ability to discriminate colors at the photoreceptor level, or the reverse, it does not matter when considering the first experience of light unless the ability to consciously perceive a full spectrum of hues was part of that first experience. Otherwise the perception of distinct hues would have been assembled later and incrementally, as the set of qualia we perceive as light was refined to implement that function. E-space can then be used as a framework for thinking both about this refinement process and about how light came to be perceived differently from other sensory modalities in the first place.

To this end, consider first an animal for which the perception of light has just emerged at a conscious level. This means at a material level that SCs capable of evoking a light experience are present. But what hue will they evoke, or, in other words, what are the characteristics of the ur-quale in terms of hue? The answer will depend on the redundancy of the system, meaning the number of active SCs required per brain to evoke a light experience. If one, then only one hue can be evoked at any one time, and this will vary between individuals in the population unless there is precise control at the SC level to ensure that each individual has replicated the same SC. But we would then need to account for why so precise a mechanism for specifying hue was already in place. Otherwise, with a less precise mode of specification, and hence a greater range of SCs at the population level, each individual would experience a different hue. Subsequently, assuming some hues or combinations of hue are better adapted for vision than others, selection would ensure those hues or combinations of hues became the population standard. More likely is a degree of redundancy, of multiple light-evoked SCs per brain, so the ur-quale for light for each individual would combine the experience of various hues, but in different ways (the point clouds would differ between individuals) so that individuals would have a similar but not identical experience. But there is then a further problem, assuming a degree of redundancy, as to whether the ur-quale for light would have been restricted to light-like sensations alone. It could instead have extended as a point cloud into regions of E-space supporting experiences that for us are associated with other sensory modalities, resulting in a mixed experience incorporating features we would recognize as belonging to those other modalities. The ur-quale for light would then differ from the pure experience of light as we perceive it, but would still have adaptive utility so long as it represented an improvement on the way light was perceived up to that point. This is because the well-known aphorism relating to vision, that “in the land of the blind, the one-eyed man is king” applies at every step in the evolutionary sequence, which is a further reminder of how distant our own consciousness today may be from subjective experience as it first emerged in evolution.

Problems like those just mentioned are simplified if we think more clearly about how an emerging ur-quale would be represented in a large dimensional space. At the level of SCs, selection will act to increase the reliability with which a given quale is evoked so as to better distinguish it from other forms of emerging experience. This means point clouds in SC-space will become more compact with time (Lacalli, 2021), which for E-space, translates into a reduction in the dispersion of point clouds across dimensions. So the end point for the evolution of light perception, at least for us, would be its restriction to just a few dimensions, namely the ones we identify as light-like based on our own experience. At issue is the starting point, of whether the ur-quale for light was initially widely dispersed across E-space dimensions or restricted to just a few. This is equivalent to asking whether, with reference to SC-space, we are dealing with a “puddle” scenario described in the paper just cited (see figure 3 in Lacalli, 2021), of an ur-quale that combines multiple forms of experience that later came to be experienced separately, or the “tree” scenario, where qualia are precisely specified from the start. Again, redundancy matters because, when it is low, individual experience would differ due to few SC- and E-space points per brain being scattered in diverse ways across the dimensions occupied by the denser point clouds mapping that same ur-quale for the population as a whole. But so long as there is some redundancy at the individual level, meaning multiple SCs per individual, the starting point for the E-space counterpart of the puddle scenario at both the individual and population level can be thought of as a diffuse point cloud with components resident in many E-space dimensions. The set of qualia we associate with a particular sensory modality, light perception in this example, would not be accidentally “discovered” by evolution, but would have been present as a sub-component in the ur-experience from the start. Evolution can then extract that subcomponent by systematically removing from the population those gene variants responsible for the SCs evoking E-space points in dimensions other than those that are light-like. The tree scenario poses more of problem, because an explanation is then required for why a particular ur-experience would already have been so precisely specified as to be restricted to few dimensions *before* selection had an opportunity to act on it as a manifestation of an emergent consciousness.

**FIGURE 3:**
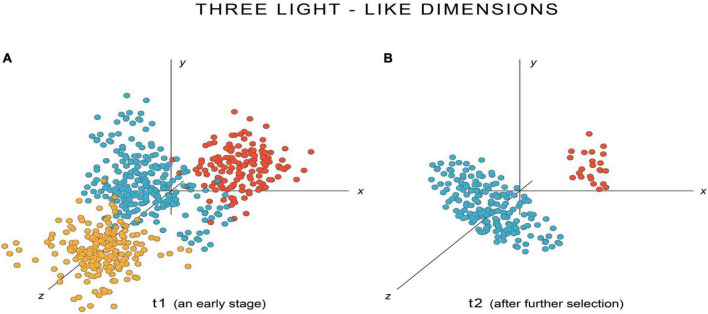
Evolving point clouds in a large dimensional version of E-space, showing three of those dimensions (*x*, *y* and *z*) that, for the sake of argument, are assumed here to map light-like experiential properties. There are of course many other dimensions that cannot be shown, and the ur-experience of light could well evoke points in those other dimensions, just as point clouds for other modalities might initially intrude, as shown here, into light-like dimensions. **(A)** Shows an early stage in the evolutionary process for a species for which consciousness is newly emergent from the preconscious condition, and **(B)** a later stage, after natural selection has had an opportunity to further refine that emergent set of experiences. The point clouds evoked in response to light stimuli are shown in blue, and those for two other modalities represented in *xyz* space in this hypothetical example, sound and odor, in orange and red, respectively. Through selection, evolution will progressively eliminate SCs evoking experiences that are maladaptive for each sensory modality, so in **(B)** point clouds evoked by stimuli other than light will have either been eliminated from these three light-like dimensions, as in the case of acoustic experience (the orange point cloud has vanished), or will be in the process of being eliminated, as here for olfactory experience (the now much smaller red point cloud). The point cloud for light experience, meanwhile, has become more compact so as to form a disc with white light at the center, as in the previous figures.

**FIGURE 4:**
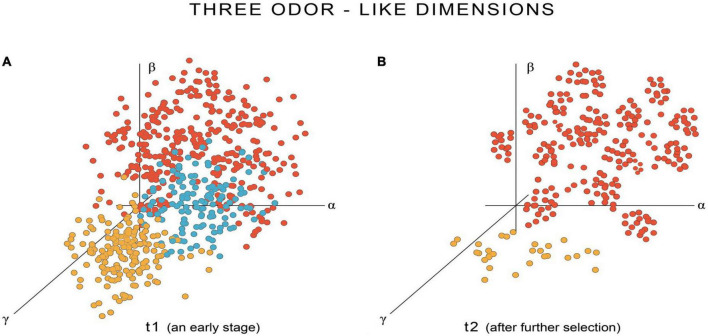
As in Figure 3, but a second set of point clouds in three other E-space dimensions (α, β, and γ) that map odor- rather than light-like properties. Blue, orange and red point clouds represent the odor-like components of ur-qualia for light, sound, and odor, respectively, as before. In **(A)** again an early stage in the evolutionary process of refinement, there is overlap between the three modalities, so that experience of both light (blue) and sound (orange) will have some odor-like properties. In **(B)** a later stage, the point cloud for odor has evolved into a clustered array, whereas odor-like features of light experience have been eliminated, and for acoustic experience, nearly so. The result overall is that the properties of qualia are sorted so as to optimize the way each is experienced while, at the same time, eliminating overlap between different categories of experience. The clustering of the olfactory point cloud in **(B)** unlike the disc used for light in the previous figure, is meant to indicate that, for olfaction, arranging for the sensations associated with related olfactory experience to be clustered, and perhaps to extend to many more dimensions, might be a way to enhance the ability to store and recall related odor experiences from memory in a systematic way. The point is made simply as a reminder that the shape and dimensionality of point clouds in E-space will likely differ, perhaps dramatically so, between different sensory modalities.

The argument is most easily appreciated by consulting Figures 3, 4, which are designed to deal with the most general case, of qualia evolving simultaneously, and of emergent SCs on which selection has only just begun to act. The SCs can then be supposed not to be as precisely specified as they eventually will be, as a consequence of selection, which translates in E-space into point clouds that are more diffuse and spread across more dimensions than they eventually will be. Figure 3 shows three coordinate axes that I will designate as representing light-like properties, though initially, as pointed out above, we could be dealing with a situation where light stimuli evoke points in other dimensions as well, perhaps many other dimensions. The figure then follows the conversion of an initially diffuse point cloud (Figure 3A, in blue), representing the ur-experience of light perception in the dimensions shown, evolving (in Figure 3B) into a flattened disk centered on the point in E-space corresponding to white light, defined as the point where all other hues are extinguished. At the same time, any other ur-experiences incorporating light-like properties will find those properties progressively eliminated. Hence, the red and orange dots in the figure, representing points in the light-like dimensions of E-space evoked by SCs in response to olfactory and acoustic stimuli, respectively, have either vanished from those dimensions at a later stage in evolution (orange dots in Figure 3), or are in the process of doing so (red dots). Figure 3 provides no indication of what hypothetically might be happening in other E-space dimensions, but Figure 4 does, for three other dimensions chosen from among those mapping odor-like properties. In this case, over a time interval comparable to that in Figure 3, it would be the odor-like properties of acoustic and light ur-experience that are progressively eliminated as olfactory experience is refined. Selection would thus be acting simultaneously in this scenario to extinguish the maladaptive light-like features from non-light experiences, and maladaptive odor-like features from non-olfactory experiences.

The point of the above line of argument is not that a justification is needed for the adaptive properties of the set of qualia employed for the perception of light, or any other sensory modality, but that there is a particular way for evolution to extract and refine those properties that allows for the most suitable set of qualia for each modality to be selected over all others. For light in particular, this would also account for how the experience of white light became the default for a combination of other hues: that if there is such a point in E-space, where all other hues are extinguished and replaced by a single hue to which they all converge, then that is where evolution will choose to center the point cloud representing light experience. The analysis does not purport to explain why there should be such a point, but so long as it exists, it explains how evolution comes to select that point over all others. It may be, however, that any closed loop in E-space generates such a point, so qualia other than light-like ones could be used for representing the light spectrum in a recursive fashion. And if so, we are no wiser than before as to whether our experience of light is uniquely suited to this purpose or not.

An objection to the above line of argument is that having emerging qualia share dimensions, and hence properties, is unrealistic if qualia are only useful as contents if they are clearly distinguishable from one another from the start, as they are today in our own consciousness. This implies precisely specified domains in SC-space as consciousness first evolved, which equates to the tree scenario referred to above, and to restricted dimensionality in E-space. But an explanation is then needed for how the SCs came initially to be so precisely specified. A possible answer, if we consider a single category of qualia evolving in isolation, is that there could be subsets of dimensions so superior in adaptive terms compared with the alternatives, that the restriction of an initially diffuse point cloud to those few dimensions occurred so rapidly as to be indistinguishable from its being precisely specified from the start. This would have consequences, especially where there is a sequence in which contents are added to consciousness as it evolves. To take a specific example, suppose light was the first sensory modality to be experienced consciously, in accord with the scenario suggested by Feinberg and Mallatt (2016). With light there is the added problem of whether we are dealing only with an experience that distinguishes consciously between light and the absence of light, or whether some form of consciously perceived 2D visual display was there from the start. But regardless, the relevant issue from an E-space perspective is that, once the point cloud evoked by light has been reduced to a suitably small number of light-like dimensions, contents added later to consciousness would evolve in a setting in which those few dimensions were already committed (i.e., occupied) and unavailable to any modality other than light perception. Hence SCs evoking a light-like experience for stimuli other than light would be strongly selected against and insignificant at a population level. The situation would be one of contents being added to consciousness in sequence, each in turn staking out a small subset of whatever uncommitted dimensions remain.

There are, however, other scenarios to be considered, including ones where there might initially have been no great advantage to selecting one set of dimensions over another for a given modality, or even to distinguish between modalities. So, for example, consider a rudimentary conscious arousal mechanism based on light and odor signals that used a nearly identical set of qualia for both. Assuming both were equally relevant signals for the initiation of a consciously controlled avoidance behavior, assigning them the same or a very similar quale would be perfectly adaptive compared with having no conscious input into the avoidance response from either modality. Differentiating the two modalities (light and odor in this example) might occur quite rapidly if there was an adaptive advantage to doing so, but there could otherwise have been a prolonged period when both were experienced in essentially the same way.

For my purposes in this account, the relative merits of any one such scenario or set of initial conditions over others is of less concern than ensuring that the broader framework, of mappings to E-space, is applicable to as wide a range of scenarios and initial conditions as possible. This would include scenarios where emotional feelings (positive and negative affect) are crucial to the narrative (e.g., Damasio and Carvalho, 2013; Solms, 2019), and modalities associated with the organs of special sense in consequence get correspondingly less attention. With the above caveats in mind, and deferring the complications inherent in special cases, I feel justified in concluding this section with the following conjecture for the general case of ur-experiences evolving together: that if it can be assumed that qualia are assigned to sensory modalities in ways that are either optimal or better than the alternatives, the most effective means of achieving this in a systematic fashion is for the ur-qualia for these modalities to begin the process as diffuse, multidimensional point clouds in E-space. This provides evolution with the widest range of options, and so avoids the problem of assigning a less-than-optimal quale by default, simply because that happened to be the way a given modality was first experienced, or because all other dimensions were already committed to other modalities. And because, for the general case, diffuse, multidimensional ur-experiences offer this advantage over narrowly specified ones, one can predict that taxa whose brains employ the diffuse option are the ones that are most likely to have survived to the present. Hence, the qualia their brains experience are more likely than not to have been selected in this fashion. For the selection process as a whole, I suggest the term “dimensional sorting” to emphasize this outcome: that an optimal sorting of qualia among available dimensions can, by this means, be achieved. In addition, and very importantly, if we can assume the sorting process occurs gradually over time, and impacts most if not all emerging contents simultaneously, this model for the process can account for the evolution of conscious experience as a balanced, unified whole. This is because contents evolving together as an ensemble are continuously being tested for their effect on the totality of experience as the sorting process proceeds.

## The experience-space/selector circuit-space relationship, and the exclusionary principle

The above analysis makes the case that divergence and optimality among qualia are facilitated by having ur-qualia occupying many E-space dimensions so that multiple distinguishable properties can be sorted among qualia. There is also then an exclusionary principle in operation, that evolution will act to prevent qualia from incorporating properties evoked by other qualia. The exclusionary principle applies in this case across all available dimensions, and should serve in practice to distribute qualia as widely as possible across those dimensions.

Divergence and the exclusionary principle also operate in SC-space, but there they act within dimensions, to maximize distance and minimize overlap between point clouds on a dimension-by-dimension basis. This distinction is worth bearing in mind when dealing with mappings from SC-space to E-space. There are cases where an isomorphic mapping is possible, for example, for closely related (i.e., homologous) qualia such as the experience of different acoustic tones (Lacalli, 2021). Minor adjustments to the SCs might in that case be sufficient to generate meaningful change within the E-space dimensions that define acoustic experience, so the mapping would be from one low-dimensional space to another. However, for change involving non-homologous forms of experience, such as a transition from an acoustic experience to one that is light-like, an isomorphic mapping seems the least likely alternative. This is because selection acts on point clouds in SC-space so as to maximize configurational differences within dimensions, but there is no corresponding benefit to reducing dimensionality *per se*. In contrast, the result in E-space will be seen predominantly in the restriction of point clouds for each category of experience to a small subset of dimensions, so the shapes of point clouds across dimensions in E-space are being changed in a fundamentally different way than in SC-space.

To go further with the evolutionary argument, there are plausible conclusions to be drawn, given suitable assumptions, as to how subjective experience would have changed as consciousness first evolved. Here I take the simplest case, of an explicitly neurophysical stance: that the evolutionary precursor of subjective experience arose from some physical consequence of neural circuit activity, which equates to “the physical” (Godfrey-Smith, 2019; Jylkka and Railo, 2019), or a neuroscientific point of view (Winters, 2021). This, in some formulations, is attributed to underappreciated properties of electromagnetic fields (McFadden, 2020; Kitchener and Hales, 2022), but regardless of details, the point is that a neurophysical stance gives meaning to the idea of redundancy, that it involves replicate circuits acting in concert. If sentience then depends on circuits exhibiting a degree of redundancy, the expectation is that those circuits would have been neither numerous nor very effective in producing sentient experience until evolution was able to further augment that experience and refine it. In other words, the initial rudiment of phenomenal experience present in the emerging conscious state would, for the individual, have been of low intensity and comparatively undifferentiated. The action of evolution would then have been twofold: to increase the intensity of the experience while, at the same time, beginning the dimensional sorting process, of extracting subcomponents and increasing their intensity individually. This would presumably have depended on increasing the redundancy of the system as a whole, because that is the only way of augmenting the raw material, at the circuitry level, on which selection acts. For the individual, there should therefore have been an increase in the intensity of experience over time from an initially negligible level, but also a transition from an undifferentiated noise-like form of experience, to one where one or more distinguishable contents emerged from this noisy background. And, for species for which consciousness is newly emergent, assuming this primarily involves qualia as opposed to more complex contents, the conscious state would be something evoked by specific stimuli, and so would have been more episodic than our own, whose complex formatted contents (e.g., vision and abstract thought) are adaptive in large part because they occupy the mind, when awake, on a more-or-less continuous basis.

## Conclusions, with caveats

This account is concerned with the evolution of consciousness, and while there are various ways of addressing the issue (e.g., Cabanac et al., 2009; Velmans, 2012; Feinberg and Mallatt, 2016; Gutfreund, 2018; Ginsburg and Jablonka, 2019; Godfrey-Smith, 2019; Black, 2021; Lacalli, 2022), the focus here is on how selection would act on the simplest contents of consciousness as they first began to evolve. Though the hard problems of consciousness enter the narrative at various points, they are not addressed directly, my view on the subject being (in accord with Block, 2009), that physics may hold the answer, but we currently lack the data and conceptual tools needed to discover that answer. But in any case, the questions one can address in evolutionary terms are less concerned with how consciousness can exist than how it got to be the way it is, and the constraints that govern its evolutionary trajectory along particular paths as opposed to others. Current theories of consciousness are diverse in their focus and claims (e.g., Atkinson et al., 2000; Van Gulick, 2018; Seth and Bayne, 2022), but the role evolution plays in determining the character of phenomenal experience is seldom dealt with as explicitly as one would like, especially by higher order theories. Yet, if we take Dobzhansky’s dictum with the seriousness it deserves, that nothing in biology makes sense except in the light of evolution (Dobzhansky, 1973), then dealing with evolutionary issues like sequence and homology is an essential part of understanding how an evolved consciousness such as ours came to be the way it is. The formulation presented here has one advantage in this respect, that it focusses attention on how the properties of experience, expressed in dimensional maps, will have changed over time, and hence on how the experiences of our distant ancestors might have differed from our own. This would include such arcane questions as to whether, for example, our species would, in its history, have had access to sensations comparable to those experienced by, say, an electric fish during an electric discharge, or a bat as it echolocates.

Certain caveats should be kept in mind with the E-space formulation as developed here. First, that it is an awkward fit for theories where qualia are not separable from consciousness as a unified whole, and hence are not individually subjects of selection (Brook and Raymond, 2021). But such theories present difficulties to an evolutionary analysis of any kind, which leaves them largely outside the concerns of evolutionary biology, and hence of this account. But even for theories of consciousness where E-space would in principle be applicable, there is a question as to how useful it is for dealing with the realm of experience. One can ask, for example, whether E-space is well founded as an analytical tool. But this is difficult to assess until we have a better understanding of the nature of the properties being mapped in this exercise including whether, for example, axes in E-space are orthogonal, as spatial dimensions would be, or can be made so. Hence, without knowing precisely what E-space axes represent, there is no guarantee that E-space has the features required for mathematical analysis, of orthogonality and continuity, or whether it has any meaning beyond being a device for ordering empirical data in graphical form.

One can nevertheless argue, at a minimum, that E-space is worth exploring if it provides insights beyond those available from more conventional forms of narrative and verbal argument. From my analysis there appear to be two such insights. First, the question of shared axes highlights the importance of ideas like those of von Békésy, uniting sound with other mechanosensations, the implication being that at least one E-space axis must be time-related. A testable prediction would then be that there are common time-related features at the neurocircuitry level shared across mechanosensory SCs, which could be proved or disproved from sufficiently detailed data on brain circuitry once such data are available. Other axes in E-space are more problematic, e.g., for light, and I can in consequence offer no useful comments on, for example, how a yellow/blue or red/green axis relates to SC structure or activity patterns. There is a further problem of hidden structure in E-space, which can again be illustrated using light perception: that using two axes to represent the observable range of hues may simply mean that a point cloud occupying more dimensions than two is experienced as if it were projected onto a 2D surface. So in Figure 3B, for example, a flat disc is used to represent light experience, yet it resides in a larger dimensional space, of three dimensions in this example, though there could conceivably be more. Our perception of hue being defined by two axes, of yellow/blue and red/green, would then, in effect, be a matter of the brain making some form of secondary coordinate transformation.

The second insight, at a more abstract level, is valid irrespective of how E-space dimensions are defined in practice. It is that unrelated sets of qualia are best represented in E-space by mapping them to non-overlapping sets of dimensions and, flowing directly from this formulation, that the assignment of qualia to sensory modalities is most efficiently achieved for contents evolving together if the respective ur-qualia are initially diffuse and multidimensional. This expands the pool of options on which evolution can draw, and is not only the better strategy from the standpoint of adaptive flexibility, but provides the best available way of conceptualizing the process by which qualia are optimized by evolution for the functions they are required to perform. I refer here to the process of dimensional sorting as described above, whereby diffuse multidimensional ur-experiences will have an evolutionary advantage over those more narrowly specialized from the start. This also resolves a philosophical question (e.g., see Majeed, 2016), of how is it that a particular assortment of conscious contents can be brought into existence. The question is inescapably an evolutionary one, for which the answer is straightforward if one assumes the process begins with ur-experiences consisting of separable components, because all that remains is for evolution to effect the separation in ways that are functionally useful.

## Data availability statement

There are no data beyond that included in this article. Further inquiries can be directed to the corresponding author.

## Author contributions

The author confirms being the sole contributor of this work and has approved it for publication.
